# BST-2 promotes survival in circulation and pulmonary metastatic seeding of breast cancer cells

**DOI:** 10.1038/s41598-018-35710-y

**Published:** 2018-12-04

**Authors:** Wadie D. Mahauad-Fernandez, Wasifa Naushad, Tyler D. Panzner, Amani Bashir, Geeta Lal, Chioma M. Okeoma

**Affiliations:** 1 Department of Microbiology and Immunology, Carver College of Medicine, University of Iowa, Iowa City, IA 52242-1109 USA; 2 Department of Pharmacology, Stony Brook University, 101 Nicolls Rd, Stony Brook, NY 11794-8651 USA; 3 Department of Pathology, University of Iowa Hospitals and Clinics, Iowa City, IA 52242-1089 USA; 4 Department of Surgery, University of Iowa, 1500 John Colloton Pavilion, 200 Hawkins Drive, Iowa City, IA 52242-1089 USA; 5 Present Address: Division of Oncology, Departments of Medicine and Pathology, Stanford University School of Medicine, 291 Campus Drive, Stanford, CA 94305 USA; 6 Present Address: Atta-ur-Rahman School of Applied Bio sciences, National University of Sciences and Technology, Islamabad, Pakistan

## Abstract

Bone marrow stromal antigen 2 (BST-2) mediates various facets of cancer progression and metastasis. Here, we show that BST-2 is linked to poor survival in invasive breast cancer patients as its expression positively correlates with disease severity. However, the mechanisms that drive the pro‐metastatic functions of BST-2 are not fully understood. Correlation of BST-2 expression and tumor aggressiveness was analyzed in human tissue samples. Migration, invasion, and competitive experimental metastasis assays were used to measure the cellular responses after silencing BST-2 expression. Using a mouse model of breast cancer, we show that BST-2 promotes metastasis independent of the primary tumor. Additional experiments show that suppression of BST-2 renders non-adherent cancer cells non-viable by sensitizing cells to anoikis. Embedment of cancer cells in basement membrane matrix reveals that silencing BTS-2 expression inhibits invadopodia formation, extracellular matrix degradation, and subsequent cell invasion. Competitive experimental pulmonary metastasis shows that silencing BST-2 reduces the numbers of viable circulating tumor cells (CTCs) and decreases the efficiency of lung colonization. Our data define a previously unknown function for BST-2 in the i) formation of invadopodia, ii) degradation of extracellular matrix, and iii) protection of CTCs from hemodynamic stress. We believe that physical (tractional forces) and biochemical (ECM type/composition) cues may control BST-2’s role in cell survival and invadopodia formation. Collectively, our findings highlight BST-2 as a key factor that allows cancer cells to invade, survive in circulation, and at the metastatic site.

## Introduction

Although metastasis is the primary cause of all cancer deaths, including breast cancer, the mediators of metastasis have not been fully discovered. In the absence of complete cure for breast cancer, it is important to identify unknown drivers of cancer progression and metastasis to address outstanding questions related to how tumor cells acquire metastatic competency to colonize a different organ with a distinct microenvironment. Such knowledge base will provide the foundation for the development of new therapeutic options for breast cancer patients. While similarities and difference exist in mechanisms of tumor growth and metastatic spread amongst different cancers, cell-autonomous roles in mediating metastasis have been described for some genes such as TGFβ^1,2^ and BST-2^3–11^.

BST-2, also known as CD317, and tetherin, was first identified as HM1.24 expressed in terminally differentiated B cells^12^. Subsequently, BST-2 was shown to possess viral tethering activity because it was discovered to be the host protein that HIV-1 viral protein U (Vpu) must counteract for viral particles to be released from infected cells^13,14^. Other viral proteins, such as chikungunya virus nsP1^15^ and influenza A virus M2^16^ have been shown to counteract BST-2, allowing viral release. Virus-mediated counteraction of host BST-2 is linked to Vpu-mediated counteraction of BST-2 activity and has been shown to regulate HIV resistance to interferon (IFN)^17,18^. Thus, in addition to tethering, BST-2 possess antiviral activity as shown by various infection models^19–22^.

BST-2 is a type II transmembrane protein composed of four domains and expressed mainly on the apical side of cells. Expression of BST-2 is regulated by both extrinsic and intrinsic stimuli, including cytokines such as interferons^20,23,24^. In different disease conditions, such as autoimmune diseases^25,26^ and different malignancies, BST-2 has been reported to be overexpressed^5,27,28^. BST-2 DNA is hypomethylated in breast cancer cells leading to its overexpression^3^. Increased expression of BST-2 in breast cancer has been shown to mediate various facets of breast cancer progression including cell adhesion, anchorage-independent growth, survival, primary tumor growth, invasion, and metastasis. The effect of BST-2 on both primary tumor growth and metastasis^4,7^ suggest that BST-2 may independently regulate both processes as inferred by Mahauad-Fernandez *et al*., in a correlation analysis^4^. Here we provide evidence that expression of BST-2 in tumors may function as an independent driver of metastasis. Using tumors from an invasive breast cancer (IBC) patient cohort, we found higher BST-2 levels in tumors that progressed beyond a localized state. Furthermore, we found that the cellular mechanism by which BST-2 exerts its pro-metastasis function includes the ability of BST-2 to promote i) formation of invasive structures, ii) survival of cancer cells in circulation, and iii) enhancement of pulmonary seeding of cancer cells and malignant growth of such cells in the metastatic site.

## Results

### BST-2 levels predict morbidity and mortality in an invasive breast cancer patient cohort

**Figure 1:**
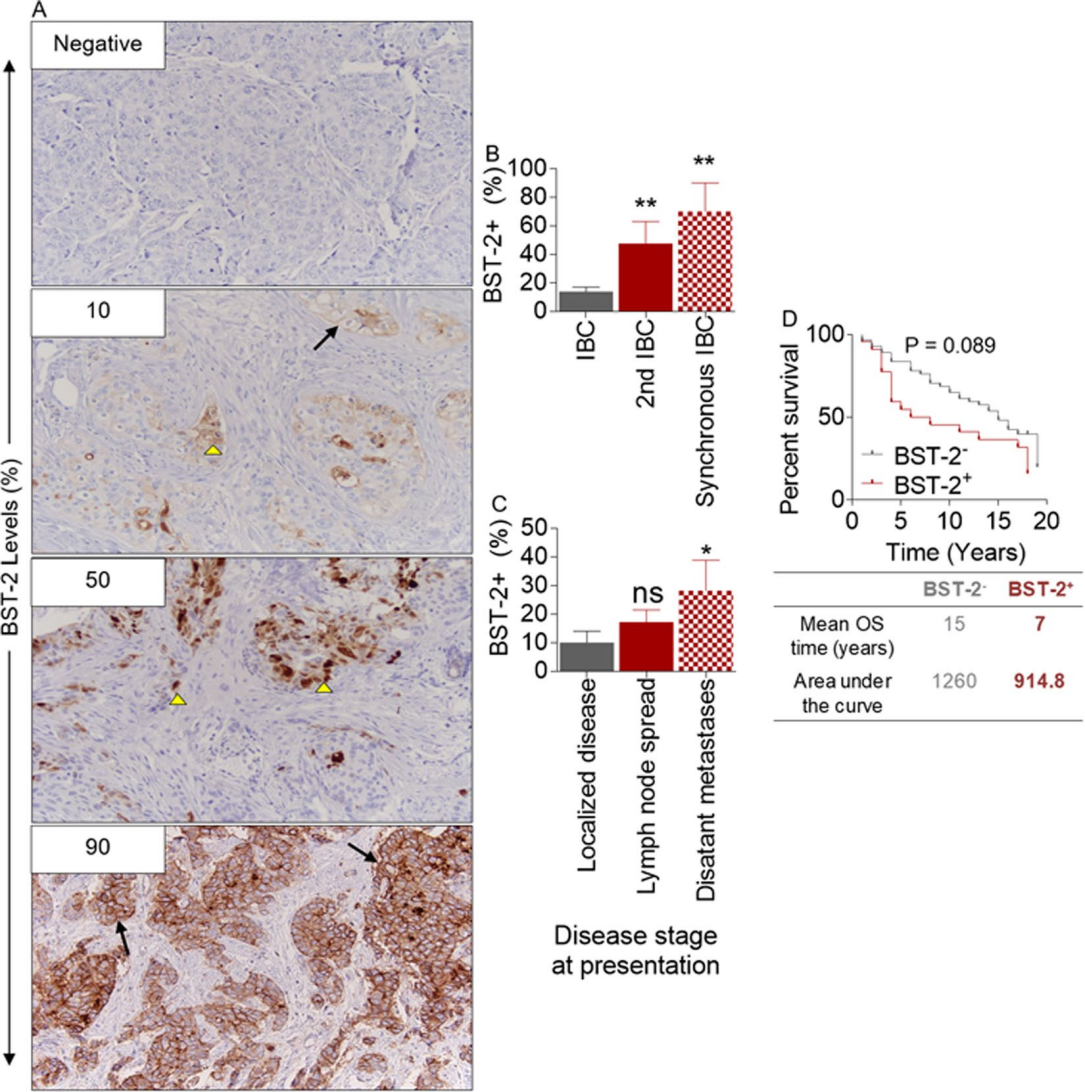
High levels of BST-2 expression correlates with disease severity in a cohort of invasive breast cancer patients: (**A**) Representative Immunohistochemistry images of BST-2 negative and BST-2 positive (≥10% staining) infiltrating ductal breast carcinomas (IBC, n = 79). Numbers correspond to BST-2 positivity represented as a percent. Magnification = 20x. Arrow heads depict cytoplasmic BST-2 staining and arrows depict accumulation of BST-2 staining at the cellular membrane. (**B**) Percent BST-2 staining in IBC tissue samples from patients with only 1 event of invasive breast cancer (IBC), patients with a second IBC after the 1st IBC (2nd IBC), and patients with synchronous breast cancer (Synchronous BC). (**C**) Percent of BST-2 staining in IBC tissue samples from patients with localized, regional or distant IBC at the time of diagnosis. (**D**) Kaplan-Meier survival plot of patients with BST-2^−^ and BST-2^+^ IBC. Patients with BST-2^−^ IBC survived for twice as long as patients with BST-2^+^ IBC. The median overall survival (OS) and the area under the curve (AUC) for BST-2^−^ and BST-2^+^ IBC patients are shown. Error bars represent SEM and numbers on top of bars correspond to p-values.

In previous studies, we utilized publicly available data sets to analyze the levels of BST-2 transcript (TCGA)^3,4^ and protein (Human Protein Atlas)^7^ to show that high levels of BST-2 positively correlates with features of aggressive breast cancer, such as survival, invasion, migration, and metastasis^3,4,7^. To link BST-2 expression in breast tumors to tumor aggressiveness, we performed BST-2 immunohistochemistry (IHC) using tumor tissues obtained from an unselected, hospital-based cohort of patients (n = 79) with invasive breast cancer (IBC) diagnosed at The University of Iowa between 1986 and 1989^29^. During this period, long-term follow–up data was collected. About 34% (27/79) of patients bear BST-2^+^ IBC; where BST-2 positivity was defined as tumors with BST-2 staining of 10% of tumor cells or higher. Where positive, membranous and cytoplasmic BST-2 staining of invasive tumor cells was noted with varying levels of intensity (Fig. 1A). In terms of disease severity, BST-2 level is highest in tumors of IBC patients with synchronous IBC followed by those that presented with a 2^nd^ metachronous IBC (Fig. 1B). In our study, we found that levels of BST-2 in tumors increased with stage of presentation from localized disease, nodal spread, and distant metastasis at the time of diagnosis (Fig. 1C). Kaplan Meier survival plots show that patients with BST-2^+^ IBC had reduced survival than patients with BST-2^−^ IBC (Fig. 1D). Although the difference in survival did not reach statistical significance, BST-2^+^ IBC-bearing patients had a median overall survival (OS) time of 7 years and an AUC of 914.8, while BST-2^−^ IBC-bearing patients had an OS of 15 years and an AUC of 1,260 (Fig. 1D). These data show that BST-2^+^ IBCs present at more advanced stages and are more aggressive than IBCs that are BST-2^−^.

### Suppression of BST-2 expression impairs migratory and invasive capability of breast cancer cells independent of cell proliferation

**Figure 2:**
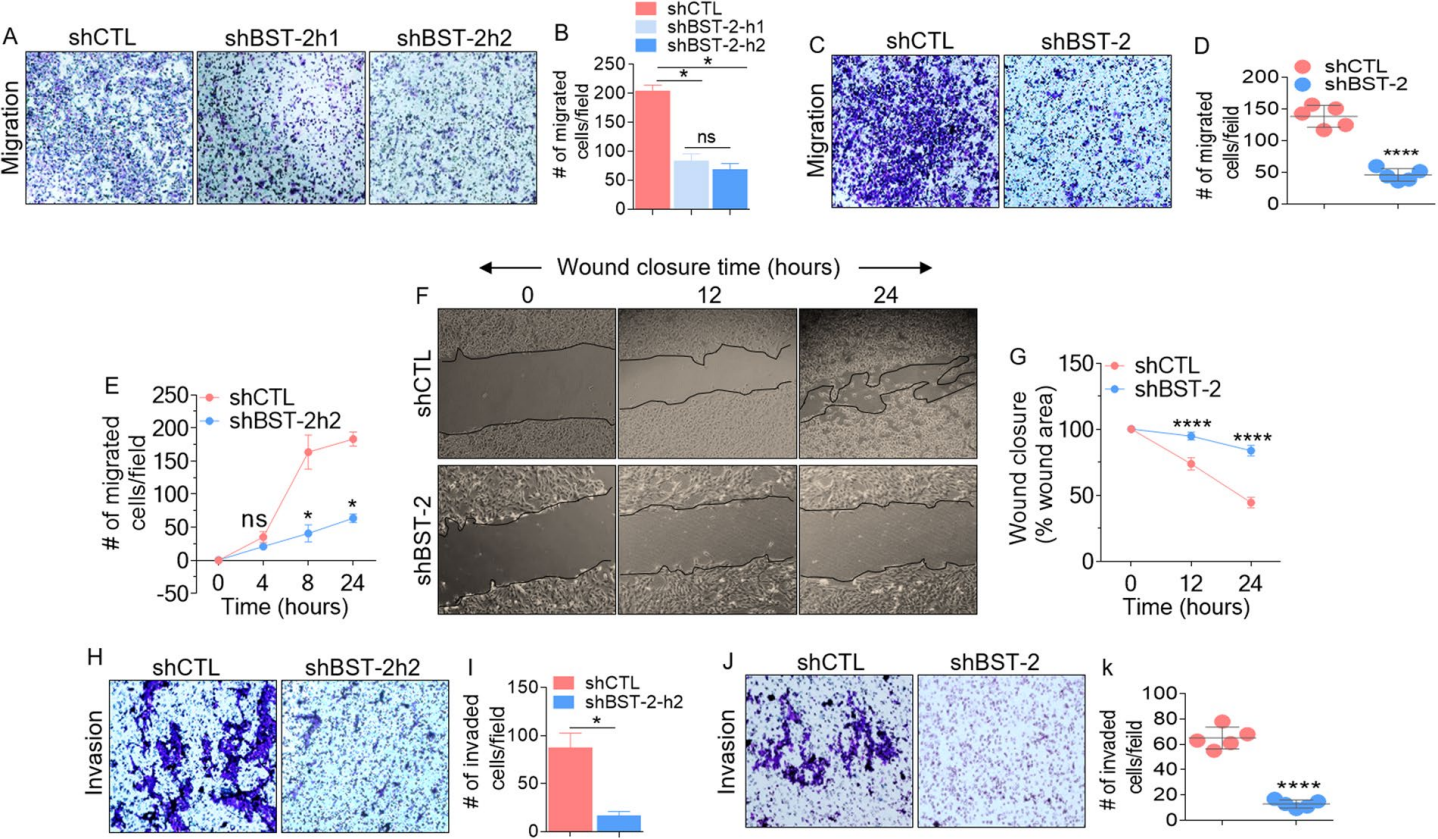
BST-2 expression promotes proliferation-independent migration and invasion of breast cancer cells: (**A**) Giemsa-stained images of migrated MDA-MB-231 cells stably expressing scramble shRNA control (shCTL) and two different BST-2-targetting shRNA sequences (shBST-2h1 and shBST-2h2). (**B**) Quantification of migrated cells in panel A. (**C**) Microscopic images of Giemsa-stained 4T1 cells and (**D**) Graphs of 4T1 migration events in panel C. (**E**) Time-dependent trans-well migration of MDA-MB-231 cells. (**F**) Images showing kinetics of 4T1 wound closure. Black lines on images depict wound border (0 h) and extent of wound closure (12 h–24 h). (**G**) Quantitative depiction of wound closure events in panel F. (**H**,**I**) Images and quantitation of MDA-MB-231 invasion events. (**J**,**K**) Representative images and quantitation of 4T1 invasion. Representative images were taken at 4x magnification. Images used for Image J quantitation of migration and invasion were taken at 20x. Events from five different fields were analyzed. Error bars represent standard deviations. Significance was taken at P < 0.05* and P < 0.0001****. Experiments were repeated more than three times with similar results.

Previous studies have shown that endogenous BST-2 promotes non-proteolytic motility (migration) and proteolytic motility (invasion) of aggressive murine breast cancer cell line 4T1 and human MDA-MB-231 cells^28^. Further, exogenous expression of BST-2 in the luminal A human breast cancer cell line MCF-7 confers migratory and invasive abilities to the cells^4,7^. However, whether the effect of BST-2 on cell motility is as a result of increased cell proliferation is unknown. We answered this question by performing 2D and 3D migration experiments in the presence of the proliferation inhibitor mitomycin C using BST-2-expressing and BST-2-suppressed 4T1^4,7^ and MDA-MB-231^28^ cells. Suppression of BST-2 expression inhibits migration of MDA-MB-231 and 4T1 cells in the presence of mitomycin C (Fig. 2A,D). To examine the kinetics of BST-2-mediated enhancement of cell migration independent of cell proliferation, we performed time course migration assay in the presence of mitomycin C at 0, 4, 8, and 24 h for MDA-MB-231 cells and 0, 12, and 24 h for 4T1 cells. MDA-MB-231 shBST-2 cells show a reduction in average migration from 8 h when compared with shCTL cells (Fig. 2E). Similarly, 4T1 shBST-2 cells were inhibited for migration as early as 12 h compared to shCTL cells (Fig. 2F,G). Noteworthy is the difference in cell morphology between BST-2-expressing shCTL and BST-2-suppressed shBST-2 cells. Following knockdown of BST-2 expression, cells adopt epithelial-like morphology, as can be observed in Fig. 2F. Furthermore, we used MDA-MB-231 and 4T1 cells to determine if the effect of BST-2 on cell invasion^4,7^ is independent of cell proliferation. Suppression of BST-2 expression in both MDA-MB-231 and 4T1 cells inhibits cell invasion in the presence of mitomycin C (Fig. 2H–K). These data confirm that BST-2 has a bonafide effect on cell motility independent of cell proliferation and that suppressing BST-2 expression in breast cancer cells can reverse this invasive phenotype.

### Suppression of BST-2 inhibits adhesion-independent survival, and subsequent migration and re-adherence of cells to target structures

To metastasize, cancer cells that acquire anchorage-independent phenotype by resisting the anoikis program have to migrate and colonize target organs. BST-2 has been shown to be critical for anoikis resistance^7^, migration, and invasion of cancer cells^4,7^. To evaluate the role of BST-2 in the migration and re-attachment of anchorage-independent cancer cells, we developed a tandem survival-migration-adhesion assay. BST-2-expressing shCTL and BST-2-suppressed shBST-2 cells were cultured under anchorage-independent conditions on a semi-solid polymer—VitroGel 3D (TheWell biosciences) for 6 days.

**Figure 3:**
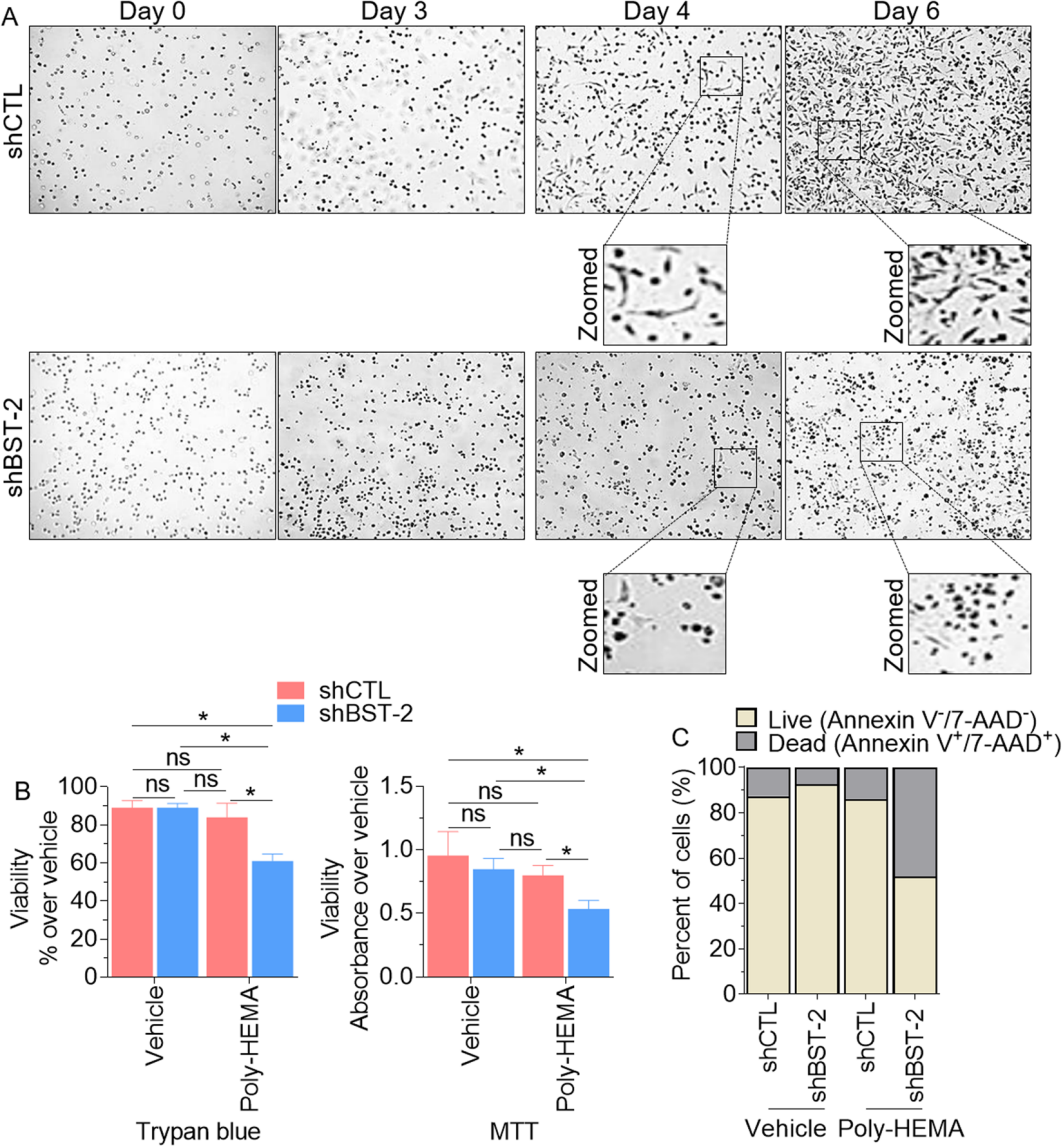
BST-2 confers adhesion-independent survival to MDA-MB-231 cells: (**A**) Representative images of cell growth and invasion using VitroGel 3D, a hydrogel similar to Matrigel, during a 6-day time period. Images show round cells which are cells in suspension that could not reach the bottom of the plate while elongated branching morphology observed in some cells depict survival in suspension and attachment to the bottom of the plate. Black boxes are enlarged areas in rows 2 and 4. (**B**) Vehicle and Poly-HEMA-suspended MDA-MB-231 shCTL or shBST-2 cells were analyzed for viability 48 h after seeding using parallel assays—Trypan Blue and MTT. (**C**) Vehicle and Poly-HEMA-suspended cells were analyzed for apoptotic markers Annexin V and 7-AAD. Live cells correspond to Annexin V^−^/7-AAD^−^ cells while dead cells were positive for one or both markers. Images were taken at 4x magnification from five different fields. Error bars represent standard deviations and significance was taken at P < 0.05*. ns = not significant.

As shown in Fig. 3A, BST-2 expression results in adhesion-independent proliferation, since shCTL cells suspended in VitroGel increased in numbers over time. The cells in VitroGel were evaluated for the ability to migrate through the hydrogel and to adhere to the culture plates. Imaging through VitroGel acquired both migrated non-adherent and adherent cells. Results show that by day 4 of culture, majority of shCTL cells migrated and subsequently adhered to the culture plates. These adherent shCTL cells display mostly elongated branching morphology (Fig. 3A, upper row, days 4 and 6 zoomed). In contrast, shBST-2 cells were predominantly round reminiscent of non-adherent morphology with few elongated cells (Fig. 3A, lower row, days 4 and 6 zoomed). These results suggest that cancer cells that lost BST-2 expression may also lose the ability to reattach to new targets following transit in circulation.

Since shBST-2 cells that are anchorage-independent (suspended in VitroGel) did not efficiently attach to culture plates, we tested their sensitivity to anoikis when cultured under adherent and anchorage-independent conditions. Thus, after 48 h of Poly-HEMA-mediated suspension of shCTL and shBST-2 cells, the cells were collected for viability analysis using the Trypan blue exclusion and methylthiazole tetrazolium (MTT) assays. Both assays indicate that viability of shBST-2 cells was significantly reduced under anchorage-independent conditions compared to shCTL cells that survived this condition (Fig. 3B), indicating that reduction in BST-2 levels may render cancer cells susceptible to anoikis as previously shown in 4T1 cells^7^. Flow cytometry analysis of the frequency of Annexin V and 7-aminoactinomycin D (7-AAD) stained cells confirmed the induction of cell death in shBST-2 cells cultured under anchorage-independent conditions (Fig. 3C). The apoptotic rates of BST-2-expressing shCTL cells were not significantly changed (12.6% versus 13.75%) after 48 h of suspension culture. In contrast, the apoptotic rates of BST-2-suppressed shBST-2 cells significantly increased (7.3% versus 48.08%) after 48 h of detachment from their extracellular environment (Fig. 3C). Together, our results suggest that reduction in BST-2 expression renders cancer cells anoikis-susceptible. Our results further suggest that loss of BST-2-conferred anoikis susceptibility may render cancer cells less invasive since the suspended cells were unable to re-adhere. It is possible that the elongation and branching observed in BST-2-expressing cells allow cells to form specialized structures for enhanced motility.

### Loss of BST-2 expression inhibits the formation of invasive structures in breast cancer cell lines

**Figure 4:**
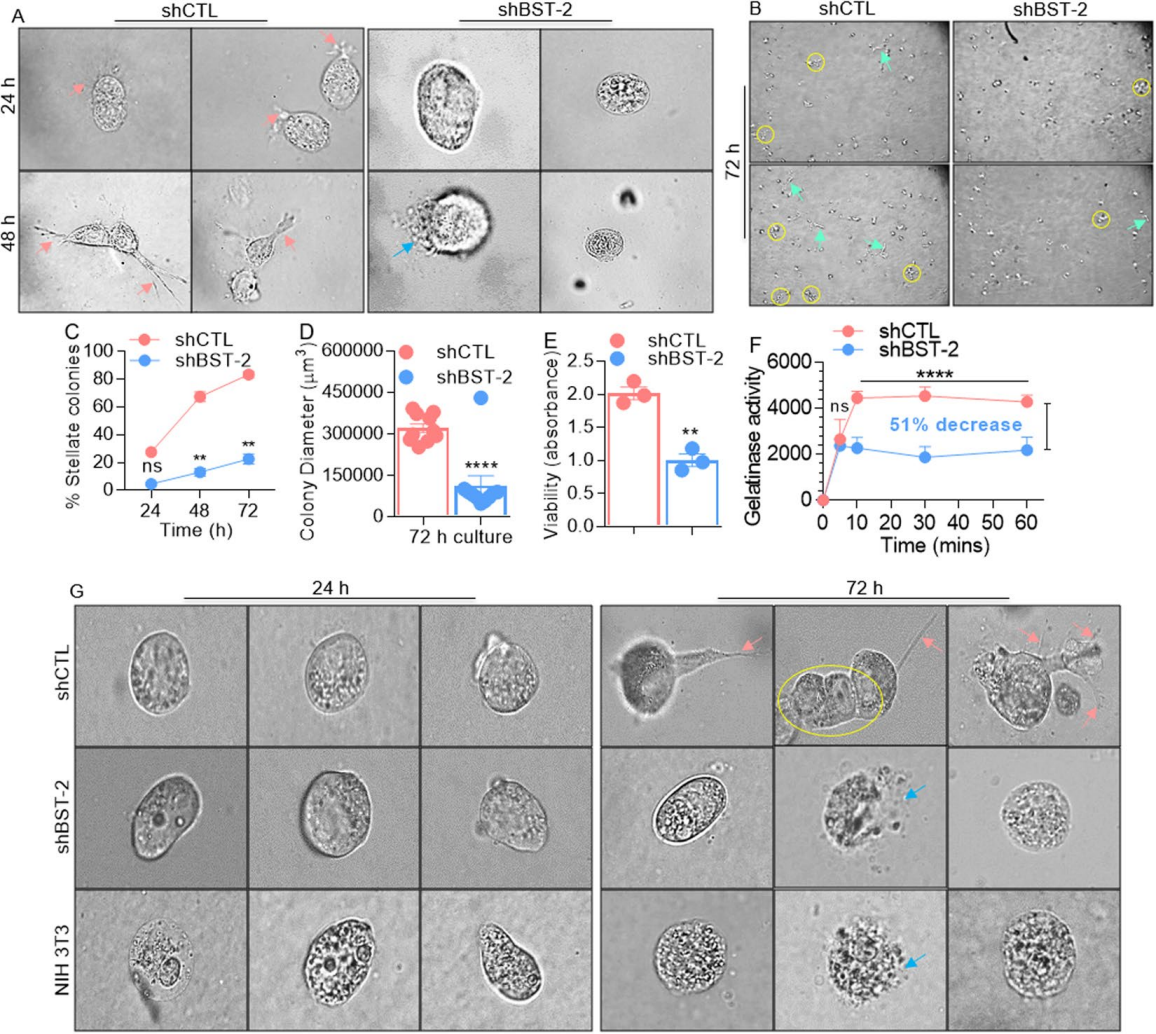
BST-2 expressing breast cancer cells form invasive (stellate) structures: (**A**) High powered microscopic images (60x) showing morphological changes and cell invasion when 4T1 cells invade 3D matrix. Following matrix embedment, shCTL but not shBST-2 cells in matrix start forming serrated invasive (stellate) protrusions by 24 h, and completely invade into the matrix by 48 h. Pink arrows show invasive structures while blue arrow shows ruptured membrane. Images were taken at stated magnification from five different fields. Olympus ix80 inverted microscope with integrated high precision focus drive was used to obtain images. (**B**) Low magnification images (4x) of 72 h matrix embedded shCTL and shBST-2 cells for quantitative evaluation of invasive structure characteristics. Cyan arrows point to invasive structures and yellow circles depict colonies. (**C**) Quantitation of the number of stellate colonies formed over time expressed as a percentage of total number of colonies per dish (invasive and non-invasive). (**D**) Quantification of colony size by measuring the diameter of 10 colonies/field. (**E**) Viability of matrix-embedded shCTL and shBST-2 cells. (**F**) Gelatinolytic activity of shCTL and shBST-2 cells. Increase in fluorescence (per time unit) between the shCTL and shBST-2 indicate enhanced catalysis. The percentage inhibition was calculated at t = 60 minutes. (**G**) High powered microscopic images (20x immersion with 6x mechanical zoom) showing cells at 0 h immediately after plating in 3D matrix and after 72 h. Pink arrows show invasive structures, blue arrow shows ruptured membrane, and yellow circle depict cell cluster. For panels C–F, error bars represent standard deviations and significance was taken at P < 0.05*, **0.01, ****0.001. n.s = not significant.

Given the importance of BST-2 in mediating anchorage-independent survival and invasion, both of which enhance metastasis, we investigated whether expression of BST-2 plays a role in the formation of invasive structures, also critical for metastasis^30^. shCTL and shBST-2 cells were plated onto Matrigel-coated cover plates as indicated^31^. shCTL cells formed colonies with stellate projection structures invading the surrounding matrix, whereas shBST-2 cells mostly grew as round spheroids without defined stellate projections. Compared to shBST-2 cells, the invasive structures can be observed by high powered microscopy on the periphery of shCTL cells as early as 24 h with complete matrix invasion at 48 h (Fig. 4A, pink arrows). In contrast, shBST-2 cells appeared rounded and by 48 h, some cells present with ruptured membrane morphology (Fig. 4A, blue arrow). By 72 h, majority of shCTL cells have invasive structures (Fig. 4B, cyan arrow heads) and also formed more and larger colonies (Fig. 4B, yellow circles) while shBST-2 cells present with dramatic loss of invasive morphology in 3D Matrigel culture at all times examined. Quantitation of the number of invasive colonies formed over time expressed as a percentage of total number of colonies per dish (invasive and non-invasive) show a significant increase in shCTL invasive structures (Fig. 4C). Additionally, by 72 h, majority of shCTL cells have larger colony diameter (Fig. 4D). Given the observed differences in cell morphology, especially with disrupted cell membrane, we assessed the viability of shCTL and shBST-2 cells suspended in Matrigel. We observed a significant decrease in the viability of shBST-2 cells (Fig. 4E). These results suggest that the effects of BST-2 on the number and length of invasive structures may reflect the promoting effect of BST-2 on spreading and invasion. Enhanced gelatinolytic activity of shCTL cells was observed in a fluorogenic dye-quenched (DQ)-gelatinase assay in which BST-2 expression increased gelatin proteolysis. At variance, suppression of BST-2 expression resulted in a 51% inhibition of gelatinolysis (Fig. 4F). To determine whether these invasive structures are unique to invasive cancer cells, we assessed the ability of NIH 3T3 cells— a non-invasive murine embryonic fibroblasts to form invasive protrusions. As shown, only 4T1 shCTL cells formed invasive structures (Fig. 4G, pink arrows) and cell clusters (Fig. 4G, yellow circle). As expected, non-invasive 4T1 shBST-2 cell and NIH 3T3 cells did not form invasive protrusions and these cells contain a population of cells that displayed ruptured membrane, indicative of cell death (Fig. 4G, blue arrows). These results indicate that BST-2 expression may enhance cancer cell invasion through increased formation of serrated protrusions and matrix degradation.

### BST-2 promotes invadopodia formation and ECM degradation

**Figure 5:**
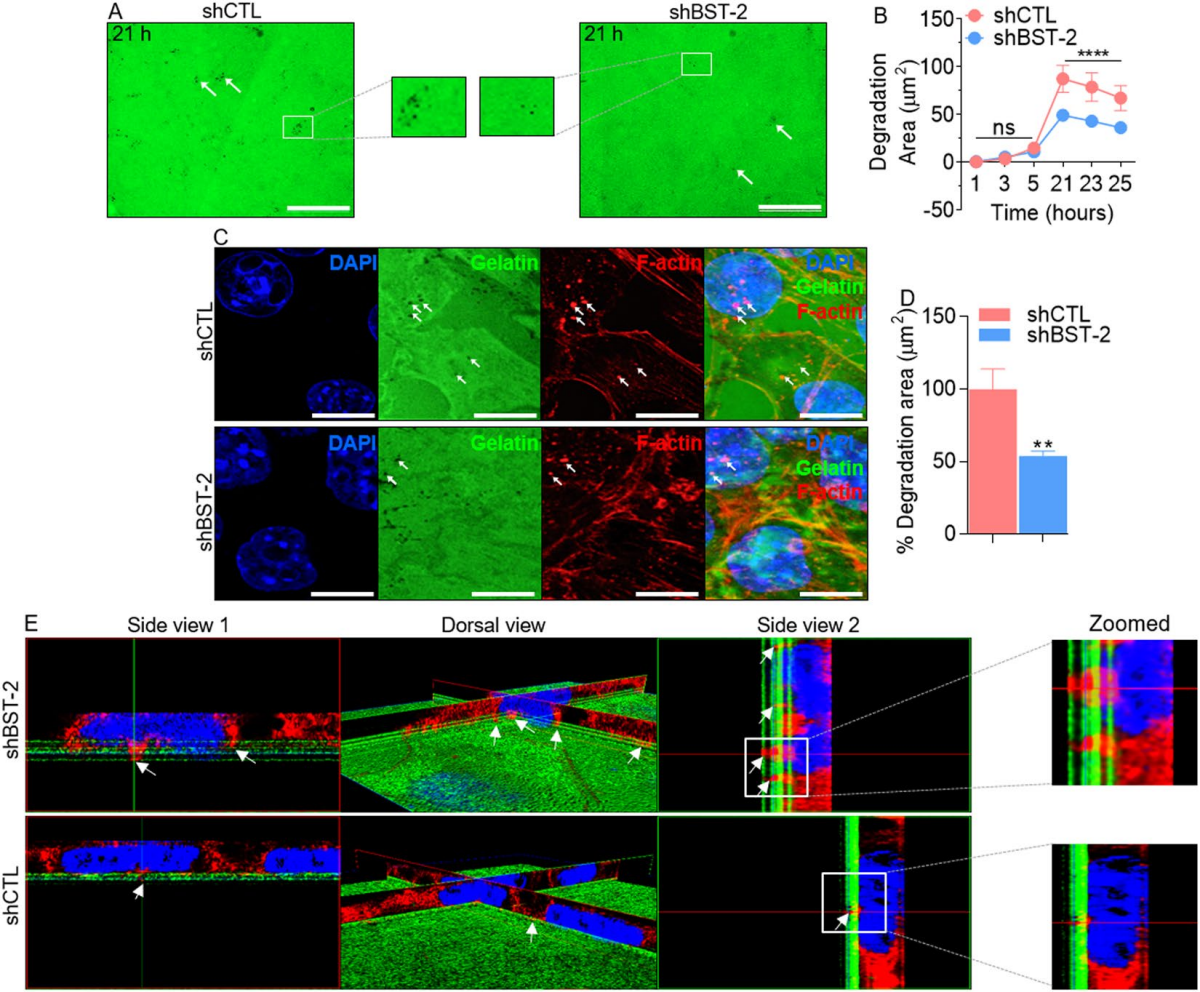
BST-2 promotes breast cancer cell-mediated gelatin degradation: (**A**) Representative images (20x) of gelatin matrix proteolysis analyzed by time-lapse microscopy. Shown is 21-hour time point. Areas of matrix degradation are black holes on the green fluorescent gelatin (white arrows). Boxed areas are magnified to show area of matrix degradation. (**B**) Area of degradation was determined from images taken from 5 fields per well for a total 12 wells (n = 60) per cell line. Degradation area was quantified using Gen5 software. (**C**) Representative confocal images (63x, 2.5 zoom) of gelatin matrix proteolysis with co-localized F-actin at 27 hours after seeding cells atop gelatin. Shown are DAPI-stained nuclei (blue), Alexa 488 fluorescent gelatin (green) with dark holes (degradation), F-actin (red), and the overlay of the three channels. Arrows indicate areas of focal matrix proteolysis at the indicated time point. (**D**) Quantification of gelatin degradation at 27-hour time point depicted as percent (%) degradation using 5 fields per well for a total 12 wells (n = 60) per cell line. (**E**) Three-dimensional volumetric reconstruction of BST-2-induced invadopodia penetrating the underlying matrix. Cells stained with Alexa Fluor 594 Phalloidin are in red and the fluorescent matrix is depicted in green. Leica SP8x confocal software was used to obtain optimal image stack. Images were deconvoluted to reconstruct a 3D image of invadopodia penetrating the underlying fluorescent matrix (white arrows). Displayed are side view 1 (Left), dorsal view (Middle), and side view 2 (Right). Boxed areas are magnified to show invadopodia penetrating the fluorescent matrix. Error bars represent standard errors of the mean and significance was taken at P < **0.01, ****0.001. Bar = 100 µm for all images.

Invadopodia are specialized degradative structures formed by tumor cells to facilitate cell intravasation and metastasis. Given the observations in Fig. 4 and the importance of BST-2 in mediating cell invasion, we investigated whether BST-2 also plays a role in invadopodia formation and ECM degradation. Fluorescent gelatin degradation assay indicates that suppression of BST-2 significantly reduced the area of gelatin proteolysis by 4T1 breast cancer cells as indicated by the loss of fluorescent signal (Fig. 5A,B). Invadopodia are obvious as focal cytoplasmic concentrations of F-actin overlap with areas of gelatin clearing—dark holes in the matrix (Fig. 5C, white arrows). The extent of gelatin degradation at the time of F-actin staining (t = 27 h) was quantified using Gen5 software for a graphic presentation as percent degradation area (Fig. 5D). Suppressing BST-2 expression decreased endogenous F-actin-enriched protrusions penetrating the underlying fluorescent matrix, compared with BST-2-expressing shCTL cells showing more F-actin-positive protrusions into the matrix (Fig. 5E). These data suggest that BST-2 expression promotes invadopodia formation, degradation of matrix substrates, and cell invasion.

### BST-2 expression is required for breast cancer cell survival in circulation and lung colonization

**Figure 6:**
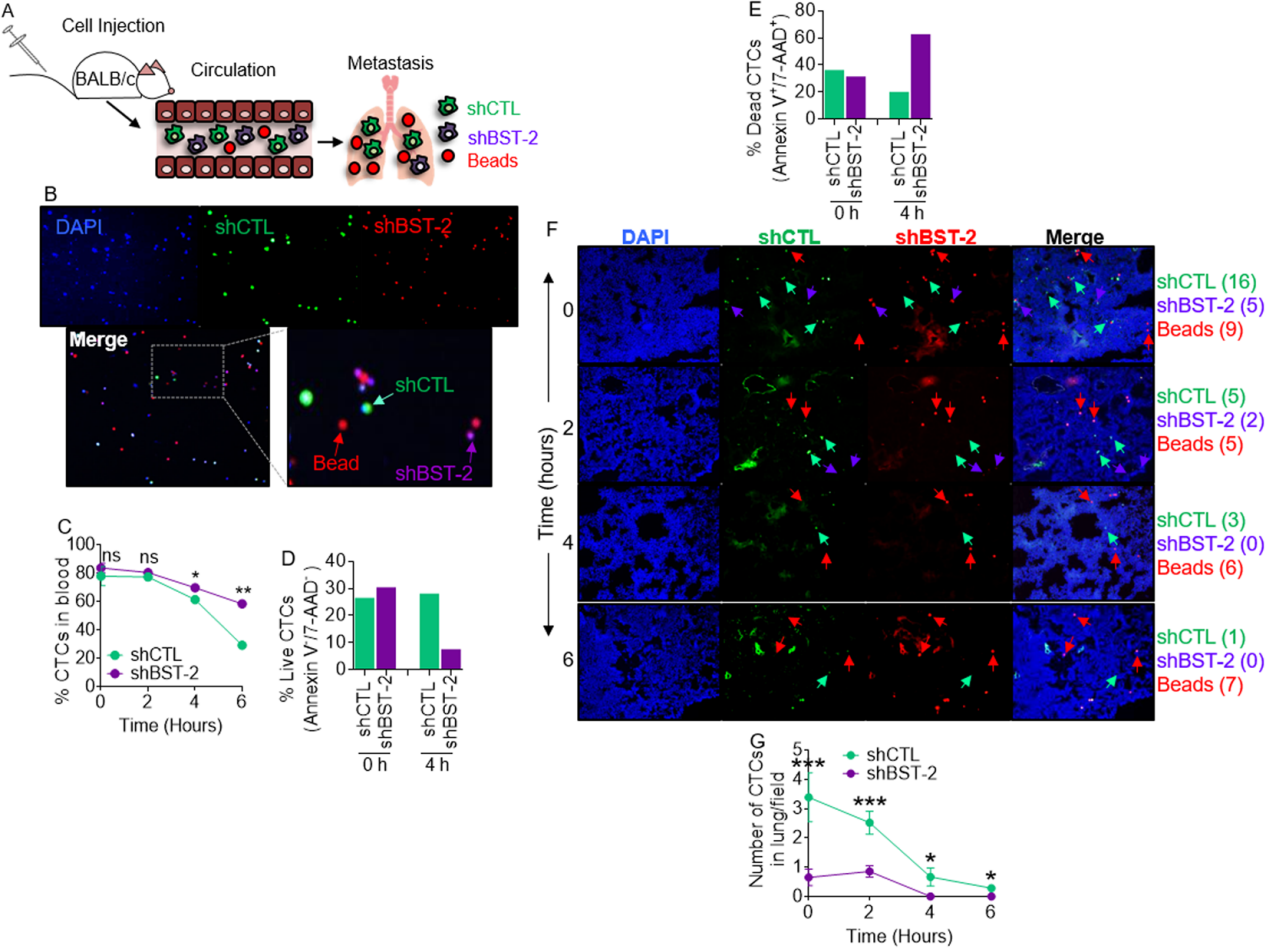
BST-2 expressing cells resist hemodynamic shear stress *in vivo*, survive in circulation, and colonize the lungs: (**A**) Cartoon of experimental set up. (**B**) Representative 10x images of cell mixture used for injections. Beads are DAPI^−^, which auto-fluoresce red (red arrows), while shCTL cells are DAPI^+^ and CMFDA^+^ (Green, green arrows), and shBST-2 cells are DAPI^+^ and CMTPX^+^ (Red, purple arrows). (**C**) Percent of CTCs present in blood at indicated times (0–6 h) after intravenous injection. Percentages were determined using flow cytometry where shCTL cells are EPCAM^+^ and CMFDA^+^ and shBST-2 cells are EPCAM^+^ and CMTPX^+^. (**D**,**E**) Percent of live and dead shCTL and shBST-2 cells at 0 and 4 h post injection. Percentages were determined using flow cytometry and staining for the early apoptotic marker Annexin V and the late apoptotic marker 7-AAD. (**F**) 10x images of lung sections from mice carrying a mixture of beads (red arrows), shCTL (green arrows), and shBST-2 cells (purple arrows) and euthanized at the indicated times (0, 2, 4, and 6 h). Merge column show all cells and beads. Numbers in parenthesis next to sample name to the right depict the number of shCTL cells, shBST-2 cells, and beads within the representative images. (**G**) Number of shCTL and shBST-2 cells per field found in the lungs at 0, 2, 4, and 6 hours. 100 beads were counted for each time point. Error bars represent SEM and significance was taken at P < 0.05*, P < 0.01**, and P < 0.001***. ns = not significant. CTCs = circulating tumor cells.

Since BST-2 promotes formation of degradative invasive structures (Figs. 4 and 5) required for cancer cell extravasation^32^; and since we have previously shown that BST-2 is an important promoter of breast cancer metastasis, we sought to define the steps at which BST-2 is involved along the metastatic cascade. We performed competitive syngeneic experimental pulmonary metastasis studies (Fig. 6A) by injecting mice via the tail vein with a mixture of 4T1 shCTL: shBST-2: beads in a 2:2:1 ratio (Fig. 6B). Beads were used for normalization of differences in injection efficiencies from mouse to mouse. Blood collected from injected mice was used to determine the relative number of shCTL circulating tumor cells (CTCs) to shBST-2 CTCs over 6 h period. We found that the number of shCTL and shBST-2 4T1 CTCs was similar at time zero and after two hours (Fig. 6C). However, by 4 h, there were more shBST-2 CTCs in blood than shCTL CTCs (Fig. 6C). Furthermore, by 4 h, the percent of live shCTL CTCs was three times higher compared to shBST-2 CTCs (Fig. 6D). At variance, there was about 20% more dead shBST-2 CTCs compared to shCTL CTCs at 4 h (Fig. 6E). These results indicate that BST-2 expression may enhance metastasis through resistance to hemodynamic stress.

Because CTCs that resisted hemodynamic shear force and immune clearance are capable of reaching and seeding secondary sites, such as the lungs, we evaluated the ability of shCTL cells to extravasate and form metastatic colonies in lungs. Thus, immediately after injection, there were about three times more shCTL cells in the lungs than shBST-2 cells (Fig. 6F,G) even though there is no difference in cell numbers (Fig. 6C) or survival in the blood (Fig. 6D,E) at this time point. The reason for this difference is unknown but it is plausible that as efficient mediators of cell to cell interaction^7^, shCTL cells may be able to evade stress and interact with other cells compared to shBST-2 cells. Interpretation of this result is convoluted by the fact that overtime, the number of shCTL cells in the lungs decreased, perhaps due to cell death or immune clearance (syngeneic model). Nevertheless, at all times, there were at least twice as many shCTL cells found in the lungs compared to shBST-2 cells (Fig. 6F,G). These data suggest that BST-2 promotes cancer cell extravasation, providing a potential mechanism for BST-2 in the invasive process during intravasation, extravasation, and colonization stages by tumor cells in the metastatic cascade.

### Suppression of BST-2 expression in cancer cells reduces metastatic lung nodules and prolongs survival of tumor-bearing mice

**Figure 7:**
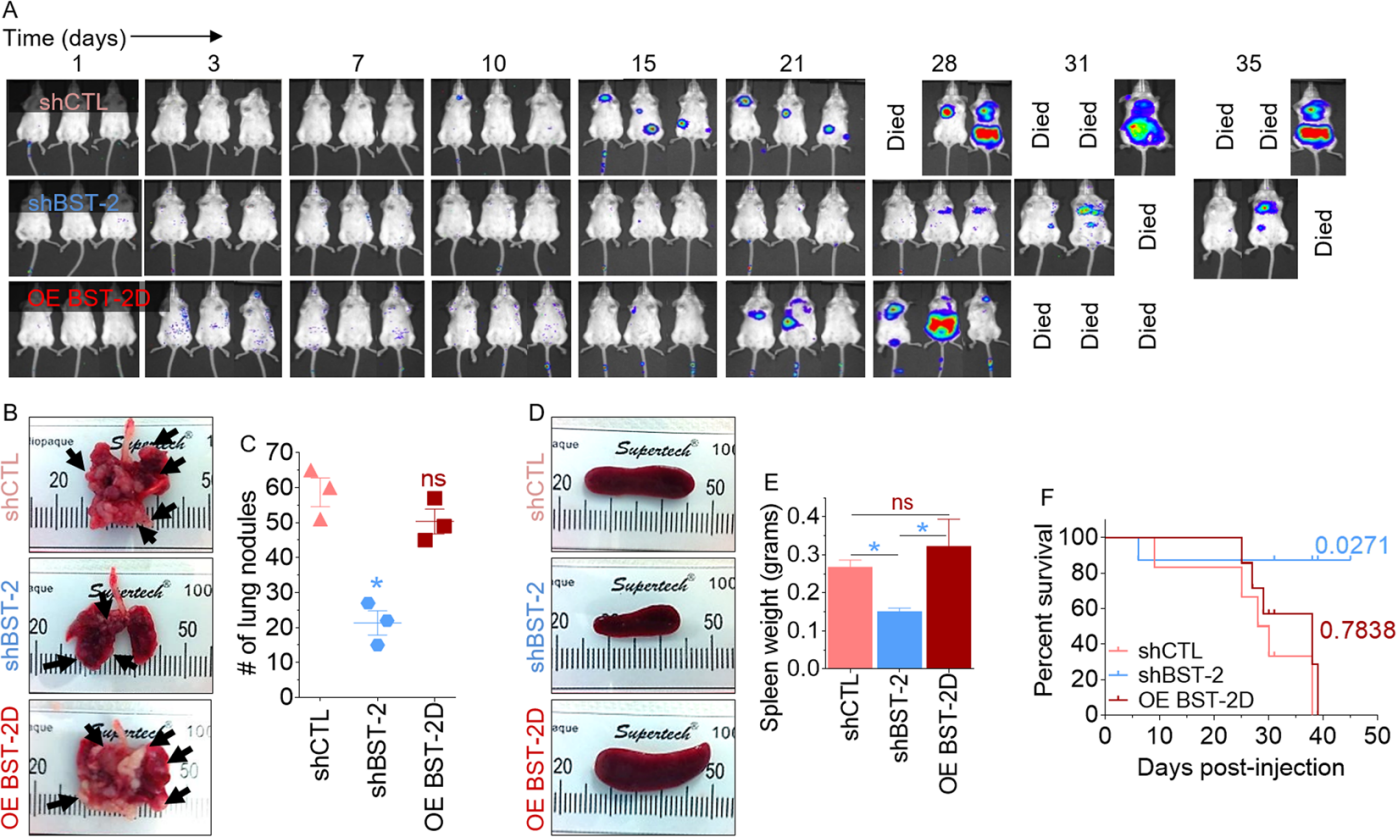
BST-2 promotes metastasis independent of primary tumor: (**A**) Representative image of metastases detected after injection of 5-week old BALB/c mice with 300,000 of 4T1 cells expressing shCTL, shBST-2 or OE BST-2D via tail vein. n = 3 for each condition. Tumor cells tracked *in vivo* with IVIS imaging at different time points. (**B**) Representative gross images of lungs showing visible pulmonary nodules (arrows). (**C**) Quantification of lung colonization events in mice described in panel B. (**D,E**) Gross images and weight of spleens of mice described in panel A. (**F**) Kaplan-Meier survival plot of mice described in panel A. Numbers are P values relative to shCTL group. Error bars represent SEM and significance was taken at P < 0.05*. ns = not significant.

Thus far, we have established that BST-2 is an important regulator of various breast cancer aggressive phenotypes and a regulator of metastatic processes. Here, we sought to determine if the observed reduction in the ability of BST-2-suppressed cells to migrate, survive in circulation, and invade *in vitro* (Figs 2–6) correlates with altered metastatic ability *in vivo*. For this purpose, we utilized the 4T1 breast cancer model in which BST-2 plays an important role in spontaneous metastasis^4,7^ to conduct experimental metastasis studies. Our 4T1 panel of breast cancer cells are well-tractable syngeneic model consisting of cells stably expressing control shRNA (4T1 shCTL) and BST-2-targetting shRNA (4T1 shBST-2), as well as 4T1 shBST-2 cells with constitutive expression of human BST-2 (OE BST-2D)^7,28^, that is resistant to shRNA directed toward the murine isoform. Using these cell lines, we performed experimental metastasis studies to examine the contribution of invasion during the extravasation and colonization steps of metastasis. Tail vein injection of 4T1 shCTL cells resulted in metastasis of cancer cells as shown by high levels of luciferase expression in mice (Fig. 7A, top panel). Dissemination of cancer cells was significantly inhibited by suppression of BST-2 expression as observed in 4T1 shBST-2 injected mice (Fig. 7A, middle panel) relative to 4T1 shCTL mice (Fig. 7A, top panel). The inhibition of metastasis was reversed by expression of OE BST-2D in shBST-2 cells (Fig. 7A, compare top and bottom panels). Additionally, suppression of BST-2 reduced rate of lung metastasis as indicated by the number of lung nodules observed in mice bearing 4T1 shBST-2 cells compared to 4T1 shCTL and 4T1 OE BST-2D bearing mice (Fig. 7B,C). Further evidence of metastatic disease is shown by analysis of spleen size. Mice bearing shBST-2 cells had normal spleen size whereas shCTL and OE BST-2D bearing mice presented with splenomegaly (Fig. 7D,E) as previously reported^4^. The morbidity that was caused by the injection of shCTL and OE BST-2D cells resulted in decreased survival of mice bearing shCTL and OE BST-2 cells compared to shBST-2-bearing mice (Fig. 7F). These results therefore demonstrate the requirement of BST-2 in lung metastasis.

## Discussion

Cancer cell migration and invasion are highly integrated and dynamic processes that precede metastasis, which is a multi-step process encompassing i) cancer cell infiltration into adjacent tissues, ii) intravasation (trans-endothelial migration) of cancer cells into vessels, iii) survival of such cells in circulation, iv) extravasation (leave the blood stream) of the cells and (v) subsequent attachment and proliferation at secondary sites leading to colonization. During cancer progression, a variety of tumor cells show resistance to detachment-induced cell death (anoikis), as well as alter their plasticity via morphological changes that may include one or a combination of collective to amoeboid transition (CAT)^33^, epithelial to mesenchymal transition (EMT)^34^, and mesenchymal to amoeboid transition (MAT)^35^. Such changes allow cells with metastatic ability to survive harsh conditions while invading incompatible distal sites. Efficient coordination of events in the metastatic cascade is necessary for successful dissemination of cancer cells because alteration in any of the key metastatic processes will eliminate and possibly destroy metastasizing cancer cells. Therefore, it is crucial to identify the factors controlling cancer cell dissemination for development of novel efficacious therapy since most cancer deaths are linked to metastasis.

BST-2 is one such factor that have been shown to be important for cancer aggressiveness, including enhanced metastasis^3,4,7–11,36–39^. However, the precise mechanism of how BST-2 functions and its contribution to various stages of metastasis are yet to be fully defined. Prior findings from our group identified BST-2 as an importatnt factor in spontaneous metastasis; which interrogates the full metastatic cascade^4,7^. This current study used pre-clinical and clinical data to provide evidence that BST-2 is directly linked to the aggressive phenotype and metastatic potential of breast cancer cells. Clinical breast tumor specimens from a cohort of invasive breast cancer patients facilitated the correlation of BST-2 levels to disease severity. Although the tumors used in our studies were from patients diagnosed with invasive breast cancer, some of which have metastasized, whether patients’ tumors disseminated through lymphatic routes and/or hematogenous routes is unknown. However, comparative analysis of patients presenting with localized disease, lymph node spread, and distant metastases using clinical specimens indicate that disease severity is in line with increasing BST-2 levels.

We demonstrate that a key effect of BST-2 expression in cancer cells is the induction of enhanced non-proteolytic and proteolytic cell motility independent of cell proliferation, as well as the ability to overcome anoikis. We previously reported that in breast cancer, BST-2 renders cancer cells resistance to anoikis through the GRB2/ERK/BIM/Cas3 pathway^7^. In addition, in nasopharyngeal carcinoma, high BST-2 expressing cancer cells can overcome anoikis via the induction of NF-kB and expression of anti-apoptotic genes^40^. While our data showing the presence of invadopodia support a role of BST-2 in cancer cell motility, this finding also reveals that BST-2 may actively control invasion events through migration-independent mechanisms that may involve stimulation of degradation of matrix substrates via metalloproteases. It remains to be determined if BST-2 is a component of mature invadopodia and which matrix-degrading factors are present in BST-2-containing invadopodia. Although the BST-2-dependent events that regulate invadopodia formation and the kinetics of elongation as well as invasive protrusion are yet to be determined, it has been shown that various proteins as well as specific phosphoinositide lipids are known to be associated with different stages of invadopodia formation and elongation in cancer cells^41^. For example, the actin cytoskeleton and filopodia- and lamellipodia-associated proteins are involved in the formation of invadopodia, whereas microtubules and vimentin intermediate filament networks play a role in elongation of invadopodia^42^. In our studies, we employed the widely used thin layer of ECM that have been coated directly onto a glass structure^31^. Although the assay clearly depicts the role of BST-2 in invadopodia formation, the glass surface may have blocked to some extent invadopodia elongation. Therefore, the effect of BST-2 on invadopodia formation may have been underestimated and requires a more appropriate physiological environment for accuracy. The fact that BST-2 mediates cell to cell and cell to ECM interactions^4,7^, cell invasion^28^, the formation of invasive structures and degradation of gelatin, suggest the possibility that BST-2 may be part of the cellular processes including CAT, EMT, MAT, ECM degradation, and basement membrane transmigration. It therefore remains to be determined whether BST-2 influences the stability of invadopodia, as well as identify the BST-2-regulated factors that mediate invadopodia formation and function.

The possible link between BST-2 and formation of invasive structures is intriguing for many reasons. Invadopodia formation has been visualized in models of breast cancer^43,44^ and these structures have been implicated as key mediators of cancer cell intravasation^45,46^. Further, invadopodia facilitates extravasation of cancer cells at endothelial junctions^32^. One of the most important characteristics for successful metastasis is for cancer cells to withstand hemodynamic (fluid) stress upon intravasation^47^. Expectedly, over 98% of cancer cells that reach blood vessels are cleared within 24 hours^48^ in part due to fluid stress. However, cancer cells have developed ways to overcome this hemodynamic stress in circulation^49^. For example, circulating cancer cells (CTCs) can cluster with each other^7,50^, with fibroblasts^51,4,7^, and with platelets^52^. CTCs that form clusters are better able to survive in circulation than CTCs that travel as singlets^50^, in part because CTC clusters travel at slower velocities and are more resistant to anoikis than CTC singlets^53^. Our finding that BST-2 expression confers survival advantage to breast cancer cells in circulation, as demonstrated using competitive syngeneic experimental pulmonary metastasis, may link BST-2 to cancer cell resistance to hemodynamic stress, and support the already known fact that BST-2 mediates interactions between cancer cells and fibroblasts and promotes cancer cell resistance to anoikis. CTCs have their own microenvironment with gradients of signaling molecules that promote cell adhesion and resistance to anoikis^53^. It is possible that *in vivo*, BST-2 may be induced by IFNs released from cancer-associated macrophages (CAMs) or myeloid cells that are part of these clusters. These gradients of signaling molecules can promote epithelial to mesenchymal transitions of some of the cancer cells found in the clusters explaining the heterocellular characteristic of CTC clusters *in vivo*^54^. In addition, as an interferon-inducible gene, BST-2 has the capacity to induce the expression of a plethora of inflammatory genes that are involved in platelet activation such as IFNβ^55^.

The efficient pulmonary seeding and metastatic progression of cancer observed with BST-2-expressing cancer cells following intravenous inoculation suggest a primary tumor-independent role for BST-2 in metastasis as well as a potential role for cancer cell autonomous BST-2 in the establishment of metastatic niche. We envision that in a tumor context, BST-2‐regulated heterotypic interactions between cancer cells and the tumor microenvironment may promote invadopodia formation, intravasation, extravasation, and metastasis of breast cancer cells. Such BST-2-regulated interactome needs to be identified for development of efficient “stop” signals for metastasis.

## Materials and Methods

### Ethics approval and consent to participate

The University of Iowa Institutional Review Board (IRB) approved the use of de-identified human tissues and clinical data while the Institutional Animal Care and Use Committee (IACUC) approved the use of mice in this study. All experiments were performed in accordance with the approved University guidelines and regulations.

### Patient cohort

Dataset and tumor bank^29^ from the University of Iowa Department of Pathology containing de-identified 150 females diagnosed with or treated for invasive breast cancer at The University of Iowa hospitals and Clinics between 1986 and 1989 was used in this study. During this time, long-term follow-up data was collected including but not limited to age at time of diagnosis, tumor histological type, tumor grade, stage at diagnosis, lymph node status, cancer recurrence, vital status, date of death, and cause(s) of death. These clinical data were obtained from the Iowa Cancer Registry Database following review of patient charts. Mortality data was obtained from the Iowa Department of Public Health. Out of 150 tissue blocks in this dataset, we used 79 blocks as the other blocks had insufficient tissues for immunohistochemistry analyses.

### Pathology analysis

All 79 tissue blocks were stained and analyzed by a single pathologist (AB) who was blinded to the clinical data. Histological type and tumor grade data was gathered using Elston-Ellis classification^29^.

### BST-2 Immunohistochemistry of human IBC tissue samples

BST2 expression in breast cancer specimens was assessed using Tissue Microarrays (TMAs) constructed from paraffin-embedded, formalin-fixed breast cancer tissue. Tissue sections were deparaffinized and rehydrated in graded alcohols. Heat mediated antigen retrieval was performed and immunohistochemical staining for BST2 was performed using Dako Envison + Rabbit Peroxidase Detection System (Dako Cytomation) using a rabbit monoclonal anti-BST2 antibody (Abcam, MO, USA). Staining was performed according to manufacturers’ protocols. Immunostains were scored according to the intensity of the staining (no staining = 0, weak staining = 1+ , moderate staining = 2+ , strong staining = 3+) and the percentage of cells staining. Staining is considered positive if equal or more than 10% (≥10%) of the tumor cells show staining.

### 3D Invasion assay

24-well cell culture inserts (Merck Millipore) were coated with 100 µl of Matrigel at 1.5 mg/ml (Sigma-Aldrich) and incubated at 37 °C for 3 hours to allow solidification. 100,000 MDA-MB-231 shCTL or shBST-2 cells were starved for 4 hours, suspended in 0.1% FBS medium and were plated on top of the Matrigel layer in 100 µl. The basal chamber of the unit was covered with 600 µl of 30% FBS medium and 5 μg/ml fibronectin (Sigma-Aldrich). Cells were allowed to invade for 24 hours at 37 °C at this point inserts were collected and processed for analysis as previously described^4,7^.

### 3D Migration assay

The migration assay was performed in a similar way as the 3D Invasion assay except that the 24-well cell culture inserts were not coated with Matrigel.

### Assessment of cell invasion and growth using VitroGel 3D

10,000 MDA-MB-231 shCTL or shBST-2 cells were plated on a semi-solid polymer called VitroGel 3D. The working solution of VitroGel 3D in RPMI was made by mixing VitroGel 3D: H_2_O at a 1:2 ratio and then mixing this solution of VitroGel 3D/water with 10% FBS RPMI at a 1:4 ratio. Cells in VitroGel 3D were added to a 96-well plate at 50 µl/well; plate was then incubated for 20 minutes at 37 °C before 50 µl of 10% FBS RPMI media was added on top of the VitroGel 3D. The ratios used here allow VitroGel 3D to be kept in a semi-solid state and thus cells in VitroGel 3D must survive being in suspension before they can invade through the gel and attach to the plastic on the bottom of the well. Cells were imaged at 0, 3, 4, and 6 days.

### Evaluation of cell survival by flow cytometry

Spheroids were formed on Poly-HEMA coated u-bottom plates as previously described^7^. Spheroids were collected and disrupted to get single cells using 1x Cell dissociation buffer according to the manufacturer’s instructions (Sigma Aldrich). Single cells were stained with the eGFP Annexin V and PI Apoptosis kit (GeneCopoeia) according to the manufacturer’s instructions. At least 10,000 events were collected per sample using the FACS caliber Flow cytometer and data was analyzed with Flowjo software.

### Induction of anoikis and assessment of cell viability

Spheroids were formed and collected as described under “Evaluation of cell survival by flow cytometry.” Single cells from the spheroids were gathered using 1x Cell dissociation buffer according to the manufacturer’s instructions. Single cells were then used in a MTT assay or in a Trypan blue viability assay as previously described^7^.

### Formation of invasive structures

Protocol for colony formation was adopted from^56^. Briefly, a 35 mm cell culture dishes with glass bottom were coated with 100 μl of Matrigel (10 mg/ml). Plates were incubated at 37 °C for 30 minutes for the Matrigel to solidify. 4T1-shCTL and shBST-2 cells were harvested, and re-suspended in 1 ml of serum free RPMI to count the cells. Cells were counted and re-suspended in 1:1 of Matrigel and serum free RPMI in a total volume of 100 µl. Then, 1500 cells (in 100 µl of 1:1 Matrigel and RPMI) were seeded on the pre-coated Matrigel plates. Cells were allowed to embed in Matrigel by incubating the plate at 37 °C for 3 hours. After 3 hours, 2 ml of complete RPMI was added to the dish. Formation of invasive structures and cell colonies were monitored by acquiring images using Olympus ix80 inverted microscope with integrated high precision focus drive or HC PL APO CS2 20x/0.75 Immersion (IMM) objective with Type F immersion oil, utilizing a 6x mechanical zoom. The percentage of stellate colonies relative to total number of colonies formed were assessed at 0 and 72 hours.

### Analysis of enzyme kinetics of conditioned medium using fluorogenic DQ™-gelatin assay

Gelatinase activity was measured using modified fluorogenic DQ™-gelatin assay according to manufacturer’s instructions. Briefly, shCTL and shBST-2 lysates were added to a 96-well plate (Corning 96 Flat Bottom Black Polystyrol). Subsequently, 15 µg/ml dye-quenched (DQ)™-gelatin substrate was added and the mixtures were incubated for 10 minutes. Thereafter, the plate was placed in the fluorescence reader (Tecan infinite 200Pro Microplate fluorescence reader) and fluorescence was measured at different time points for 2 h at 37 °C (excitation = 485 nm and emission 530 nm). DMSO which prevents MMPs from binding to gelatin was used as negative control.

### Fluorescent Gelatin Coating

Glass-bottomed 24-well cell culture plates (Mattek Corp.; catalogue # P24G-1.0-13-F) were incubated in 1N HCl for 10 minutes at room temperature, then washed with Dulbecco’s PBS. Wells were incubated with 50 µg/ml poly-L-lysine (Sigma, P8920) in PBS for 15 minutes follow by additional Dulbecco’s PBS washes. Fluorescent Pig Skin gelatin, 5 mg (Thermofisher, catalog #G13186) was dissolved in 5 ml ddH_2_O according to the manufacturer’s instructions. A 5% (w/w) gelatin/sucrose solution was prepared as well. Both gelatin solutions were heated at 37 °C, mixed 1:1, then returned to the water bath for an additional 10 minutes. This working solution was then removed from the water bath and centrifuged at 10,000 × g for 5 minutes to remove aggregates. Coverslip well bottoms were coated with 90 µl of pre-warmed 1:1 gelatin working solution and allowed to sit at room temperature for 10 minutes. Gelatin was cross-linked with glutaraldehyde (Sigma, catalog #340855), prepared in a 0.1% solution in cold PBS for 20 minutes on ice, then washed with Dulbecco’s PBS, and reduced (15 minutes) with 5 mg/ml of sodium borohydride. This was followed by extensive Dulbecco’s PBS washing, then sterilization with a 30 minute incubation with 70% Ethanol using aseptic technique in a Class II Biosafety Cabinet (BSC). Plates were stored aseptically in the dark at 4 °C with 10x Penicillin-Streptomycin in PBS until used.

### Gelatin Degradation Assay

Gelatin-Coated Mattek plates were rinsed with Dulbecco’s PBS, follow by 1 hour incubation in Phenol-free RPMI media (Gibco, catalogue #11835) in the cell culture incubator for equilibration. Both shCTL and shBST-2 cells were trypsinized, counted, and equivalent numbers (120,000) of cells were plated in each well. The culture plates were placed in the Lionheart FX Live-cell imaging microscope, equipped with full temperature and CO_2_ control to maintain 37 °C and 5% CO_2_. Z-stack images were acquired 1, 3, 5, and 21, 23, and 25 hours after plating for 5 fields of view per well at 20x magnification. The total area of gelatin degradation was quantified using Gen5 software (BioTek). The values over time for 5 fields of view for each well were averaged together, which were then averaged again across the 12 wells for shCTL and shBST-2 cells.

### Immunofluorescence staining, immunofluorescence microscopy, and image analysis

Cells of interest (shCTL and shBST-2) seeded atop gelatin-coated Mattek plates for 27 hours were fixed for 20 minutes with room temperature 4% PFA. After fixation, cells were washed with Dulbecco’s PBS and permeabilized with 0.1% Triton X-100 for 15 minutes, followed by additional Dulbecco’s PBS washes. Cells were then incubated with Alexa Fluor^TM^ 594 Phalloidin (Life Technologies, catalog #A12381) diluted 1:200 in PBS for 1 hour. Cells were then washed with Dulbecco’s PBS and incubated with 300 nM DAPI (Sigma, catalogue #D9542) for 10 minutes in the dark. Z stack images were acquired using the Leica SP8x Microscope using the 60x/1.40 oil objective with 2.5 zoom. Z stacks were deconvoluted by Huygens Essential software and analyzed by Leica SP8x software.

### Mice

Age-matched (5-week-old) BALB/cAnNCr female mice were purchased from Harlan and house at the University of Iowa vivarium.

### Intravenous injections

Mice were injected intravenously via the tail vein with 300,000 4T1 shCTL, shBST-2 or OE BST-2D cell in 100 µl. Prior to luminescence imaging, mice were anesthetized and injected intraperitoneally with D-luciferin (Sigma-Aldrich) as previously described^7^. The Xenogen IVIS three-dimensional optical imaging system (Caliper Life Sciences) was used to image injected mice. Mice were sacrificed when they became moribund or at the end of the experiment. At necropsy, pulmonary nodules were manually quantified.

### Competitive experimental pulmonary metastasis

8-week-old BALB/cAnNCr females were purchased from Harlan and used in competitive experimental metastasis studies. Mice were injected via the tail vein with 2 × 10^6^ 4T1 shCTL, 2 × 10^6^ shBST-2, and 1 × 10^6^ beads in 200 µl of PBS. shCTL and shBST-2 cells were previously stained with CellTracker Green CMFDA and CellTracker Red CMTPX respectively following manufacturer’s (Thermo Fisher) instructions. Following injection, mice were euthanized at 0, 2, 4, and 6 hours at which point blood was collected in heparin-coated tubes (Microtainer) and lungs were collected, inflated with 1:1 OCT:PBS and flash frozen in OCT blocks.

### Assessment of circulating tumor cell (CTC) numbers in the blood

Blood was collected at 0, 2, 4, and 6 hours post injection from mice injected with shCTL, shBST-2 and beads as described previously. Blood was processed using Histoplaque-1077 (Sigma Aldrich) to collect PBMCs and CTCs according to the manufacturer’s instructions. Isolated cells were re-suspended in FACS buffer and stained with either PE-conjugated anti-mouse EPCAM antibody (BioLegend) or appropriate immunoglobulin Gs (IgGs) for 1 hour at 4 °C. Cells were washed once and stained with 7-AAD viability dye for 15 minutes. Using FACS Calibur flow cytometer, 100,000 events were collected per sample. FACS data were analyzed by Flowjo software.

### Evaluation of CTC survival by flow cytometry

Following PBMC isolation as described in the previous paragraph, cells were stained with PE-conjugated anti-mouse EPCAM antibody, washed once and re-suspended in Annexin V binding buffer and then cells were stained with Annexin V and 7-AAD using the eGFP Annexin V and PI Apoptosis kit (GeneCopoeia). At least 100,000 events were collected per sample using the FACS Calibur flow cytometer and data were analyzed using FlowJo software. Percent of live cells was determined by gating for EPCAM^+^, Annexin V^-^ and 7-AAD^−^ cells while dead CTCs were determined by gating for EPCAM^+^, Annexin^+^, and/or 7-AAD^+^ cells.

### Cryo sectioning, fixation, and mounting of lung slides

Frozen blocks of lungs were cut using a Thermo Microm HM 550 Cryostat (Thermo Scientific). 20 µm-thick slices were collected every 200 µm for a total of 20 slices per lung and they were fixed in 4% PFA for 30 minutes. Following fixation, slides containing lung slices were washed twice with 1x PBS and mounted using DAPI-containing VectaShield (Vector Laboratories Inc.). Lung slices were then covered with coverslips. Lung slides were imaged at 10x using a Nikon Eclipse Ti microscope adjusted with a Nikon digital sight camera. Images were processed, and lung associated shCTL, shBST-2, and beads counted using ImageJ software.

### Identification of shCTL and shBST-2 cells in the lungs

To identify and distinguish shCTL, shBST-2 and beads from each other, we used fluorescent and bright field imaging. In fluorescent mode, shCTL cells appear DAPI+ and CMFDA+ (green) and shBST-2 cells appear DAPI+ and CMTPX+ (red). Beads which are not cells are DAPI- with auto-fluorescence in the red channel. In bright field mode, beads appear light blue. Thus, to identify shCTL and shBST-2 cells, they must fulfill the following criteria: 1) be DAPI+ , 2) be green (shCTL) or red (shBST-2), 3) be smaller than beads, and 4) be present within the alveolar tissue. With this criteria in mind, we quantified shCTL and shBST-2 cells that were present in the lung at different time points until we counted a total of 100 beads per time point.

### Statistics

The GraphPad Prism software was used to perform statistical analysis of significant differences. We used unpaired t tests assuming Gaussian distribution with Welch’s correction to analyze all data except patient cohort data. For the later, we used unpaired t tests assuming both populations have the same standard deviation. Error bars correspond to standard error of the mean (SEM) for all data except for transcription data in which case we used standard deviation. To analyze Kaplan-Meier survival plots, we used the Gehan-Breslow-Wilcoxon test. A probability (P) value equal to or lower than of 0.05 was considered significant.

## Electronic supplementary material

Representative time lapse movies of invadopodia formation

Representative time lapse images of invadopodia formation
