# Supplementary material for: BST-2 promotes survival in circulation and pulmonary metastatic seeding of breast cancer cells
Source: Sci Rep. 2018 Dec 4;8:17608. doi: 10.1038/s41598-018-35710-y (PMC6279795; doi:10.1038/s41598-018-35710-y)
Supplement: Supplementary file 2 — Representative time lapse images of invadopodia formation [file 41598_2018_35710_MOESM2_ESM.pdf]

BST-2 promotes survival in circulation and pulmonary metastatic seeding of breast cancer cells

Wadie D Mahauad-Fernandez[1], Wasifa Naushad[1], Tyler D Panzner[2], Amani Bashir[3], Geeta Lal[4], Chioma M Okeoma[*1]

[1]Department of Microbiology and Immunology, Carver College of Medicine, University of Iowa, Iowa City, IA 52242-1109, USA

[2]Department of Pharmacology, Stony Brook University, Stony Brook, NY 11794-8651, USA

[3]Department of Pathology, University of Iowa Hospitals and Clinics, Iowa City, IA 52242-1089, USA

[4]Department of Surgery, University of Iowa, 1500 John Colloton Pavilion, 200 Hawkins Drive, Iowa City, IA 52242-1089, USA

Wadie D Mahauad-Fernandez - Email: wmahauad@stanford.edu

Present address: Division of Oncology, Departments of Medicine and Pathology, Stanford University School of Medicine, 291 Campus Drive, Stanford, CA 94305, USA

Wasifa Naushad - Email: wasifa.naushad@gmail.com

Present address: Atta-ur-Rahman School of Applied Bio sciences, National University of Sciences and Technology, Islamabad, Pakistan

Tyler D Panzner - Email: tyler.panzner@stonybrook.edu

Amani Bashir - Email: bashira@healthcare.uiowa.edu

Geeta Lal - Email: geeta-lal@uiowa.edu

*Corresponding author - Chioma M Okeoma

Email: chioma.okeoma@stonybrook.edu

Present address: Department of Pharmacology, Stony Brook University, 101 Nicolls Rd, Stony Brook, NY 11794-8651, USA

Key words: BST-2, Extracellular matrix, Invadopodia, breast cancer, metastasis, cell survival

Running title: Silence BST-2 to reduce metastasis

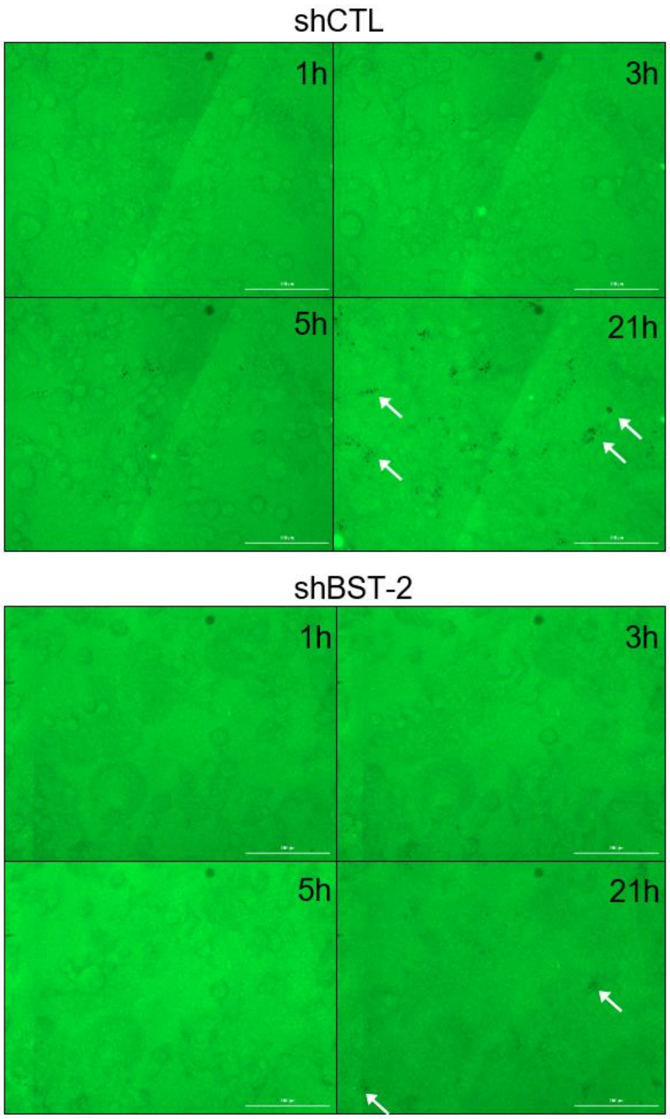

Supplemental Figure 1. Representative time lapse images of invadopodia formation: Images of green fluorescent gelatin were taken at 20x magnification with the Lionheart FX Live-cell imaging microscope. Bar = 100 µm. Arrows point to areas of matrix proteolysis (black holes).
